# Radiotherapy enhances CXCR3^high^CD8^+^ T cell activation through inducing IFNγ-mediated CXCL10 and ICAM-1 expression in lung cancer cells

**DOI:** 10.1007/s00262-023-03379-6

**Published:** 2023-01-23

**Authors:** Chih-Liang Wang, Ai-Sheng Ho, Chun-Chao Chang, Zong-Lin Sie, Cheng-Liang Peng, Jungshan Chang, Chun-Chia Cheng

**Affiliations:** 1 Division of Pulmonary Oncology and Interventional Bronchoscopy, Department of Thoracic Medicine, Chang Gung Memorial Hospital, Linkou, Taoyuan 333 Taiwan; 2 Division of Gastroenterology, Cheng Hsin General Hospital, Taipei, 112 Taiwan; 3 Division of Gastroenterology and Hepatology, Department of Internal Medicine, Taipei Medical University Hospital, Taipei, 110 Taiwan; 4 Division of Gastroenterology and Hepatology, Department of Internal Medicine, School of Medicine, College of Medicine, Taipei Medical University, Taipei, 110 Taiwan; 5 TMU Research Center for Digestive Medicine, Taipei Medical University, Taipei, 110 Taiwan; 6 Radiation Biology Research Center, Institute for Radiological Research, Chang Gung University, Taoyuan, 333 Taiwan; 7 Institute of Nuclear Energy Research, Atomic Energy Council, Taoyuan, 325 Taiwan; 8 Graduate Institute of Medical Sciences, School of Medicine, College of Medicine, Taipei Medical University, Taipei, 110 Taiwan

**Keywords:** CD8^+^ T cells, CXCR3, CXCL10, IFNγ, ICAM-1, Lung cancer, PD-L1, STAT3, Radiotherapy

## Abstract

Radiotherapy (RT) not only damages tumors but also induces interferon (IFN) expression in tumors. IFNs mediate PD-L1 to exhaust CD8^+^ T cells, but which also directly impact tumor cells and potentially activate anti-tumor immune surveillance. Little is known about the contradictory mechanism of IFNs in regulating CD8^+^ T-mediated anti-tumor activity in lung cancer. This study found that RT induced IFNs and CXCL9/10 expression in the RT-treated lung cancer cells. Specifically, RT- and IFNγ-pretreated A549 significantly activated CD8^+^ T cells, resulting in significant inhibition of A549 colony formation. RNAseq and consequent qPCR results revealed that IFNγ induced PD-L1, CXCL10, and ICAM-1, whereas PD-L1 knockdown activated CD8^+^ T cells, but ICAM-1 knockdown diminished CD8^+^ T cell activation. We further demonstrated that CXCR3 and CXCL10 decreased in the CD8^+^ T cells and nonCD8^+^ PBMCs, respectively, in the patients with lung cancer that expressed lower reactivation as co-cultured with A549 cells. In addition, inhibitors targeting CXCR3 and LFA-1 in CD8^+^ T cells significantly diminished CD8^+^ T cell activation and splenocytes-mediated anti-LL/2shPdl1. In conclusion, we validated that RT suppressed lung cancer and overexpress PD-L1, CXCL10, and ICAM-1, which exhibited different roles in regulating CD8^+^ T cell activity. We propose that CXCR3^high^CD8^+^ T cells stimulated by CXCL10 exhibit anti-tumor immunity, possibly by enhancing T cells-tumor cells adhesion through CXCL10/CXCR3-activated LFA-1-ICAM-1 interaction, but CXCR3^low^CD8^+^ T cells with low CXCL10 in patients with lung cancer were exhausted by PD-L1 dominantly. Therefore, RT potentially activates CD8^+^ T cells by inducing IFNs-mediated CXCL10 and ICAM-1 expression in tumors to enhance CD8^+^ T-tumor adhesion and recognition. This study clarified the possible mechanisms of RT and IFNs in regulating CD8^+^ T cell activation in lung cancer.

**Supplementary Information:**

The online version contains supplementary material available at 10.1007/s00262-023-03379-6.

## Introduction

Lung cancer is the most common type and the leading cause of cancer-related deaths worldwide, 80% of which are non-small cell lung cancer (NSCLC). In Asia, patients with lung adenocarcinoma have dominantly appeared in non-smokers with EGFR-positive and exon19 deletion and exon21 L858R mutation [1]. Therefore, targeted therapies like tyrosine kinase inhibitors are promising to eradicate lung cancer in clinical practice [2]. For other types of lung cancer without dominant gene mutations, targeted therapies are not suggested.

Immunotherapies are currently applied in clinical practice and result in good therapeutic outcomes in patients with lung cancer [3]. For patients without adequate application of targeted therapies, immunotherapies are an option for lung tumor treatment. One of the immunotherapies is based on the activation of CD8^+^ T cells through blocking immune checkpoints by antibodies targeting PD-1 and CTLA4 in CD8^+^ T cells [4], enhancing CD8^+^ T cells to recognize tumor cells by TCR-MHCI:neoantigens interaction and secrete granzymes and perforin to elicit tumor cell apoptosis and death [5]. In principle, it is well known that tumor cells express PD-L1 and bind to PD-1 to exhaust CD8^+^ T cells [6–8]. One of the factors to stimulate PD-L1 expression in tumor cells is interferon (IFN) [9]. Therefore, tumor cells are considered to escape CD8^+^ T cell attack in the inflammatory tumor microenvironment due to overexpression of PD-L1 derived by IFNs. However, besides induction of PD-L1 in the inflammatory microenvironment, IFNs also mediate MHCI expression in tumors [10, 11] that theoretically enhances recognition and activation of CD8^+^ T cells. Meanwhile, it has been reported that IFNs enhance anti-tumor immune responses [12], which are positively associated with better patient survival in several cancers [13, 14]. The observation is contradictory for IFNs in tumor treatment. Therefore, the investigation of the detailed mechanism is required to understand the role of IFNs in the tumor microenvironment.

IFNs are not only secreted by immune cells, but also by virus-infected cells. Short viral DNAs or RNAs specifically activate STING signaling pathways, resulting in IFNs and interferon-stimulated genes (ISGs) overexpression [15, 16]. Literature has indicated that radiotherapy (RT)-treated tumors also mediate the expression of type I IFNs [17]. The possible mechanism is that RT-damaged DNA induces consequent activation of cGAS-STING signaling pathways, resulting in type I IFNs and ISGs expression and secretion [18, 19]. In practice, literature has indicated that RT enhances the homing rate of immune cells to the tumor microenvironment [20, 21] that is stimulated by IFNs and ISG CXCL9/10 [22, 23]. However, we know that IFNs induce PD-L1 expression in tumors [24, 25], which possibly exhausts CD8^+^ T cells. Current studies have demonstrated that RT improves the anti-tumor efficacy of clinical immunotherapies targeting PD-1 [26–28]. Indeed, overexpression of PD-L1 is positively correlated with tumor immunotherapies [29], but controversial is how PD-L1, which is already abundantly expressed in tumors, continues to express and increase anti-PD-1 immunotherapy. We propose that the phenomena may be derived from other molecules mediated by IFNs-mediated other gene expressions, such as MHCI for neoantigens presentation [30] or ICAM-1 for T lymphocyte adhesion [31].

We previously demonstrated that RT induced IFNs in lung A549 cancer cells [22] and HCC PLC5 cells that further induced CD8^+^ T cell activation [32]. However, we found that RT also induced PD-L1 expression in PLC5 cells [32]. We, therefore, hypothesize RT induces not only PD-L1 but also CD8^+^ T cell-recognized molecules for further activation of CD8^+^ T cells in the tumor microenvironment. This study intended to investigate the RT effect on lung cancer cells to explain the clinical phenomena that RT induces immune system activation in tumor patients, particularly for CD8^+^ T cells since CD8^+^ T cells are the main eradicator of tumors.

## Materials and methods

### Healthy volunteers and patients with lung cancer

Blood samples were acquired from healthy volunteers (*N* = 14) and patients with late-stage lung cancer (*N* = 6) before clinical therapies at Chang Gung Memorial Hospital, Linkou, Taiwan. The human study was approved by the regulatory authorities and Institutional Review Boards at Chang Gung Memorial Hospital, Linkou, Taiwan (202102475B0, 3 March 2022). Signed and informed written consent were obtained from all participants, and the research was performed under the relevant guidelines and regulations.

### Cell culture

The A549 human lung cancer cell line used in this study was purchased from the American Type Culture Collection (ATCC, Manassas, VA, USA). A549 was reauthenticated through short tandem repeat profiling (Applied Biosystems, Waltham, MA, USA) before this study. LL/2 mouse lung cancer cell line was purchased from Bioresource Collection and Research Center, Taiwan. The two cell lines were both cultured in Dulbecco’s Modified Eagle’s Medium (DMEM). The tumor cells cultured in the medium were supplied with 10% fetal bovine serum (FBS) and 1% penicillin–streptomycin (P/S). All cells were cultured at 37 ºC with 5% CO_2_.

### Splenocytes

Male C57BL/6 mice were purchased from the National Laboratory Animal Center, Taiwan. The 5-week-old mice were housed in a 12 h-light cycle at 22 °C. The animal studies were approved by the institutive ethical review committee at Chang Gung University, Taiwan. Splenocytes were purified from a grinded mouse spleen passing through a cell strainer by washing the cells with 5 ml of RPMI twice and removing red blood cells with its lysis buffer (Sigma-Aldrich, St. Louis, MO, USA). Splenocytes were collected and cultured in RPMI with 10% FBS and 1% P/S for further experiments.

### Cell viability

The WST-1 assay (Sigma-Aldrich, St. Louis, MO, USA) was used to determine the cell viability in tumor cells treated with RT and immune cells according to the manufacturer’s protocol. In brief, 1 × 10^3^ cells tumor cells with indicated treatments were seeded in a 96-well plate and 4-times repeats were conducted for each experiment group. After 48 h incubation, the medium was replaced with a 90 μL of RPMI medium mixed with 10 μL of WST-1 reagent for 2 h incubation. The viability was detected by measuring the absorbance at 450 nm minus a reference wavelength at 650 nm.

### Immunoprecipitation and detection of IFNα and IFNγ

2 × 10^6^ A549 cells in a 6-well plate were treated with 0 and 10 Gy irradiation and cultured at 37 °C for 24 h. The culture medium was collected and centrifuged at 2000 rpm for 10 min to remove cell debris. The supernatant was consequently incubated with 1 μg/mL of rabbit-anti-IFNα (Elabscience, Houston, Texas, USA) and rabbit-anti-IFNγ antibodies (ABclonal, Woburn, MA, USA) at 4 °C overnight. The antibodies were consequently captured by 0.2 mg/mL of Dynabead-Protein A (Life Technologies, Waltham, Massachusetts, USA) at room temperature for 2 h. The Dynabeads were pulled down by a Sample Magnetic Rack and the supernatant was collected. To make sure that IFNα and IFNγ were expressed in the medium, Western blots were used for detecting IFNα and IFNγ. In brief, Dynabeads were washed with 1 mL of PBS buffer and resuspended in 100 μL of RIPA buffer (50 mM Tris, 150 mM NaCl, 0.5% sodium deoxycholate, 1% IGEPAL, and 0.1% SDS) with 20 μL of sixfold SDS sample buffer (Alfa Aesar, Heysham, Lancashire, UK). The same antibodies targeting IFNα and IFNγ were used to detect the targets in Western blots.

### Colony formation

A colony formation assay was used to evaluate the inhibitory effect of RT and PBMCs in the selected tumor cell lines. In brief, 1 × 10^3^ tumor cells pre-treated with RT and 10- or 20-fold numbers of PBMCs were seeded in a 24-well plate. Colony formation was observed under an ECLIPSE Ti2 inverted microscope (Nikon, Tokyo, Japan) after 7 day incubation, and the cell numbers per view were measured by a particle analysis using ImageJ (National Institutes of Health, Bethesda, Maryland, USA, https://imagej.nih.gov/ij/ accessed on 5 July 2021).

### Quantitative polymerase chain reaction (qPCR)

Individual total RNA from 5 × 10^5^ tumor cells, 5 × 10^5^ CD8^+^ T cells, or 5 × 10^6^ non-CD8^+^ PBMCs with indicated treatments was isolated. The procedure is described previously [33]. In brief, cells were collected and lysed in 200 μL of TRIzol reagent (Thermo Fisher Scientific, Waltham, MA, USA) mixed with 80 μL of 1-bromo-3-chloropropane. After 13,000 rpm centrifugation for 15 min at 4 °C, RNA remained in 200 μL of the aqueous phase and was transferred to a fresh tube and precipitated by adding 200 μL of isopropanol. The pellet was collected after 13,000 rpm centrifugation for 10 min at 4 °C and washed with 200 μL of 70% ethanol. Furthermore, the pellet was dissolved in RNase-free water after air-drying and the concentration was subsequently detected using a SpectraMax iD3 Multi-Mode Microplate Reader (Molecular Devices, San Jose, CA, USA). Consequently, the complementary DNA was synthesized from 1 μg of RNA using a ToolScript MMLV RT Kit (Biotools, New Taipei, ROC) according to the manufacturer’s protocol. qPCR was performed using an SYBR Green-based system (Thermo Fisher Scientific, Waltham, MA, USA). The expression of specific genes was quantified based on a 3-times repeat with normalization to GAPDH. The primer sequences for the reaction are listed in Table S1.

### RNAseq and bioinformatics analysis

RNAseq was performed to uncover the differential genes in A549 cells treated with 20 ng/mL of IFNγ for 2 h using HiSeq 4000 with paired-end 150 bp sequencing. The differential genes are shown in Table S2. Kaplan–Meier plotter (https://kmplot.com/analysis/) was used to analyze the correlation between the mRNA expression and survival probability, and the correlation between genes and anti-PD-1 (PD-L1) immunotherapeutic survival rate in patients with lung cancer based on GEO, EGA, and TCGA databases. cBioPortal (https://www.cbioportal.org/) was used to analyze the correlation between PD-L1 and ICAM-1 in patients with lung cancer.

### Flow cytometry

In brief, individual 5 × 10^5^ A549 cells after treatments for 24 h were collected in 100 μL of DMEM medium and incubated with anti-PD-L1-PE and anti-ICAM-1-APC (Biolegend, San Diego, CA, USA) for 30 min at room temperature. Subsequently, 900 μL of phosphate-buffered saline was added and mixed. The cells were then analyzed using a FACSCalibur Attune N × T Flow Cytometer (Invitrogen, Waltham, MA, USA). The same steps were followed to detect the CXCR3 (CD183), LFA-1 (CD11/CD18), and PD-1 (CD279) expression in CD8^+^ T cells of the PBMCs which were stained with anti-CD45-Pacific blue, anti-CD3-APC/Cy7, anti-CD8-PE/Cy5.5, anti-CD4-PE, anti-CXCR3-Alexa700, anti-LFA-1-FITC, and anti-PD-1-APC (Biolegend, San Diego, CA, USA).

#### Isolation of peripheral blood mononuclear cells (PBMCs) and CD8^+^ T cells

PBMCs from blood samples were isolated using centrifugation by Ficoll-Paque PLUS media (Cytiva, Marlborough, MA, US) as previously described [34]. In short, 10 mL of whole blood from each healthy donor and 3 mL of whole blood from each patient were centrifuged at 1200 rpm for 30 min without braking to isolate 3 mL of buffy coats. The isolated buffy coats were mixed with 4 mL of PBS buffer and loaded onto the 4 mL of Ficoll solution and consequently for 2000 rpm gradient centrifugation for 20 min at room temperature. PBMCs on the interface between the plasma and Ficoll media were collected, and erythrocytes that remained in the PBMCs were lysed with 1 mL of RBC lysis buffer (Sigma-Aldrich, St. Louis, MO, USA). After PBS wash, the PBMCs were resuspended in RPMI culture media and counted for further assays. The CD8^+^ T cell and non-CD8^+^ PBMCs were isolated using a MACS CD8^+^ T Cell Isolation Kit, human (Miltenyi Biotec, North Rhine-Westphalia, Germany) following the manufacturer’s manual. CD8^+^ T cells were positively harvested by a specific CD8 antibody labeled with magnetic microbeads retained in a binding column placed in a MACS separator. Non-CD8^+^ PBMCs passed through the column were collected, which theoretically contain dendritic cells (DCs), macrophages, natural killer cells (NK), and CD4^+^ T cells.

#### Enzyme-linked immunosorbent assay (ELISA) for granzyme B (GZMB) and CXCL10 measurement

Commercial Human Granzyme B ELISA kit (ABclonal, Woburn, MA, USA) and CXCL10 ELISA kit (RayBiotech, Norcross, GA, USA) were used to determine the GZMB and CXCL10 concentration, respectively, in the medium of PBMCs co-cultured with A549 cells. For GZMB measurement, 5 × 10^6^ PBMCs were cultured with 5 × 10^5^ A549shLuc and A549shPD-L1 for 24 h, and the supernatant of the culture medium was collected after 1200-rpm centrifugation for 5 min. For CXCL10 measurement, 5 × 10^6^ PBMCs from a healthy volunteer or a patient with lung cancer were cultured with 5 × 10^5^ A549 for 24 h, and the supernatant of the culture medium was collected after 1200-rpm centrifugation for 5 min. Each 100 μL of the supernatant medium was loaded with pre-coated anti-GZMB antibody or anti-CXCL10 antibody in a 96-well microplate and the consequent steps, including washing, antibodies incubation, and absorbance detection, are completed according to the manufacturer’s instruction.

#### Gene knockdown

Gene knockdown was conducted using a short-hairpin RNA (shRNA)-expression lentivirus system that contains the specific shRNA in the pLKO.1-puro vector generated by 293 T cells. The plasmids were purchased from the National RNAi Core Facility of Academia Sinica, Taipei, Taiwan. In brief, 293 T cells (70% confluence) cultured in DMEM culture medium were transfected with 4 μg of pLKO.1 vector, 1 μg of the envelope plasmid pVSV-G, and 3.6 μg of the packaging plasmid pCMVDR8.91. The plasmids were pre-incubated with 6 μL of JetPRIME (Polyplus-transfection, New York, NY, USA) for 20 min at room temperature and consequently added to 293 T cells. The cultured medium was substituted with a fresh culture medium after 24 h and further incubated for 48 h. The virus solution was collected and stored at − 80 °C. A549 cells cultured in 80% confluence were infected with the prepared lentivirus for 24 h. The cells were then changed with a DMEM medium containing 4 μg/mL of puromycin, which was harvested after obtaining stable cells.

#### Statistical analysis

Statistical analyses were performed using GraphPad Prism V8.01 (GraphPad Software, Inc., CA, USA). Significant differences for comparison of every two groups were assessed by unpaired two-tailed Student’s t-test, whereas more groups were evaluated using one- or two-way ANOVA followed by Tukey’s or Sidak’s multiple comparison test. Pearson’s correlation was used to calculate the correlation coefficient of CXCR3 between CD4^+^ T and CD8^+^ T cells in healthy volunteers and patients with lung cancer, in which *r* = − 0.3 ~ 0.3: poor correlation; *r*: 0.3 ~ 0.6 and − 0.3 ~ − 0.6: medium correlation; *r* = 0.6 ~ 0.9 and − 0.6 ~ − 0.9: high correlation; *r* = 1 and − 1: complete correlation. A *p*-value less than 0.05 was considered statistically significant.

## Results

### Radiotherapy enhances healthy PBMCs to suppress lung cancer in vitro

To investigate whether RT suppresses lung cancer and enhances immunity to eradicate tumors, the cell viability and colony formation were measured in the lung cancer A549 and LL/2 cells treated with X-ray irradiation. We found that radiotherapy by exposure of at least 10 Gy and 4 Gy, respectively, significantly suppressed A549 and LL/2 cell viability (Fig S1A) and colony formation (Fig S1B). We further found that healthy PBMCs isolated from a healthy volunteer suppressed 10 Gy of RT-treated A549 cell viability compared to untreated A549 cells (Fig S1C). In addition to confirm the observation and exclude PBMCs-to-A549 suppression was mediated by allograft rejection, the experiment was repeated and validated in 4 Gy of RT-treated LL/2 treated with syngeneic graft splenocytes isolated from a C57BL/6 mouse. The results revealed that splenocytes suppressed RT-treated LL/2 cell viability compared to untreated LL/2 (Fig S1C).

### Radiotherapy increases IFNs and augments PBMCs for suppressing A549 in vitro

**Fig. 1:**
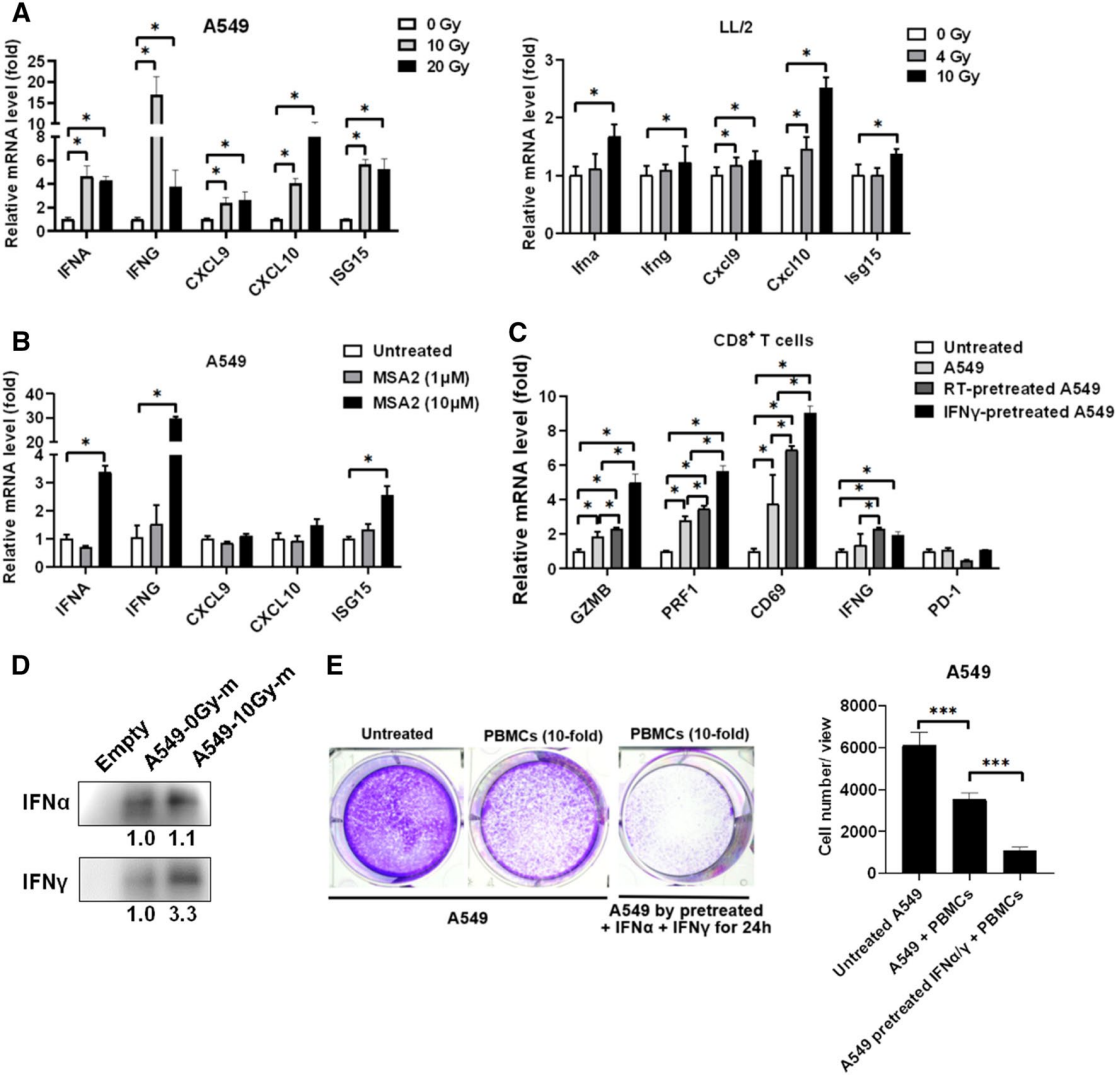
Radiotherapy (RT) increases IFN expression that stimulates cancer cells to reactivate CD8^+^ T cells. **A** IFNs, including IFNA and IFNG, and ISGs, including CXCL9, CXCL10, and ISG15, were detected in A549 and LL/2 cells treated with irradiation and (**B**) 1 μM and 10 μM of MSA2, an agonist of STING, for 24 h. **C** A549 cells pretreated with 10 Gy of irradiation and 20 ng/mL of IFNγ were incubated with healthy PBMCs for 24 h. Consequently, the cytotoxic markers granzyme B (GZMB), perforin (PRF1), and activation markers CD69, IFNG, and exhaustion marker PD-1 were detected using qPCR in the CD8^+^ T cells isolated using a MACS CD8^+^ T Cell Isolation Kit. **D** IFNα and IFNγ levels were detected in the cultured medium of A549 treated with 0 Gy and 10 Gy of irradiation using Western blots after IFNα and IFNγ captured by co-immunoprecipitation. (E) Meanwhile, colony formation was used to assess the anti-A549 activity of PBMCs in the A549 pretreated with 20 ng/mL of IFNα and IFNγ for 24 h compared to untreated A549. *p < 0.05

To investigate the detailed mechanism of RT of healthy PBMCs and splenocytes suppressing A549 and LL/2, respectively, the genes including IFNs and ISGs were detected using qPCR in the RT-treated tumor cells. We found that RT increased IFNα, IFNγ, CXCL9, and CXCL10 expression in A549 cells in a dose-dependent manner, whereas ISG15 was used as a radiotherapeutic marker (Fig. 1A). In addition, we confirmed the results in LL/2 cells (Fig. 1A). Moreover, we demonstrated that MSA-2, a STING agonist, significantly induced IFNα, IFNγ, and ISG15 expression in A549 cells (Fig. 1B), which revealed that RT induced IFN expression through the STING-mediated signaling pathway. Therefore, the additional IFNγ were incubated with parental A549 cells for 24 h, which were consequently re-incubated with a cultured medium containing healthy PBMCs for another 2 h, and CD8^+^ T cells were then isolated and analyzed using qPCR. We found that A549 pre-treated with 10 Gy of RT and 20 ng/mL of IFNγ significantly stimulated CD8^+^ T cells to increase cytotoxic markers GZMB, PRF1, and activation markers CD69, IFNγ compared to the untreated A549 group (Fig. 1C). To validate that RT induces IFN secretion, the cultured medium was collected and analyzed for IFNα and IFNγ levels in A549 cells treated with and without 10 Gy of RT. We found that secreted IFNα and IFNγ in the RT-treated A549 medium were higher than that in A549 without treatment (Fig. 1D). In addition, A549 pre-treated with IFNs (20 ng/mL of IFNα and IFNγ mixture) for 24 h incubation increased PBMCs to suppress A549 colony formation (Fig. 1E). The results suggest that IFNs alter gene expression in A549 cells to activate CD8^+^ T cells in vitro.

### Radiotherapy induces PD-L1, CXCL10, and ICAM-1 expression through autocrine IFNs stimulation in lung cancer

**Fig. 2:**
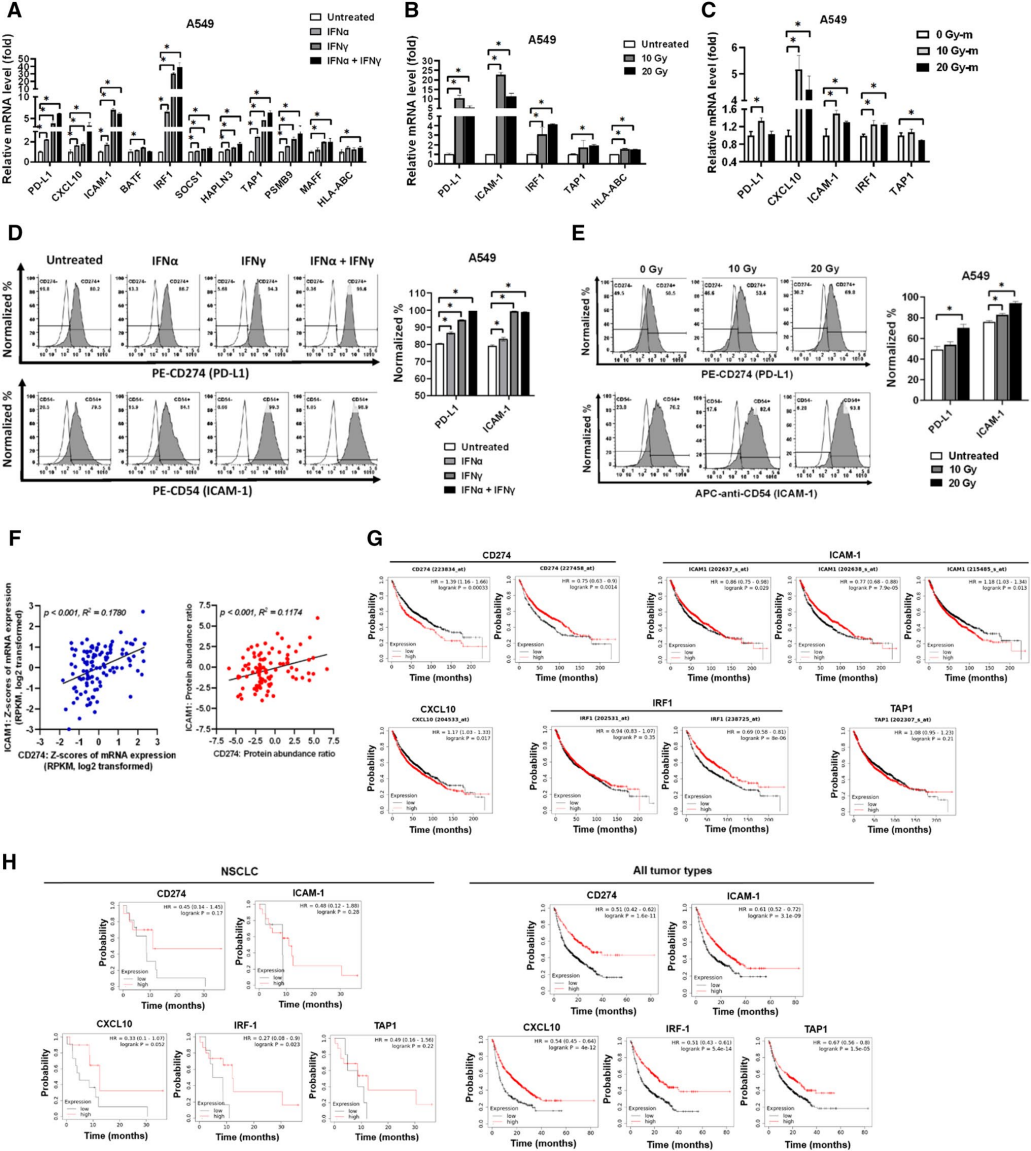
Radiotherapy and IFNγ individually increase PD-L1 and ICAM-1 expression in A549 cells, which correlates positively and is associated with better immunotherapeutic efficacy. **A** The gene expression of the up-regulated differential genes and antigen-presented HLA-ABC were validated using qPCR in A549 treated with 20 ng/mL of IFNα and IFNγ for 2 h. **B** The four dominant IFNs-mediated genes, including PD-L1, ICAM-1, IRF1, TAP1, and HLA-ABC were also validated in A549 treated with 10 Gy and 20 Gy of irradiation post 24 h cultured. **C** The cultured medium was collected in A549 treated with 10 Gy or 20 Gy irradiation (called 10 Gy-m or 20 Gy-m), which was consequently incubated with parental A549 for 2 h. The Five dominant IFNs-mediated genes were also validated using qPCR in A549 treated with 10 Gy-m and 20 Gy-m compared to 0 Gy-m. **D** To validate the protein levels of PD-L1 and ICAM-1, flow cytometry was used in A549 treated with IFNα and IFNγ for 24 h and (**E**) in A549 exposed with 10 Gy and 20 Gy of irradiation. **F** The correlation was analyzed between PD-L1 and ICAM-1 in patients with lung cancer based on the database in cBioPortal (https://www.cbioportal.org/). **G** The up-regulated differential genes selected from the RNAseq analysis of IFNγ-treated A549 (Table S2) were analyzed for the correlation with survival probability in patients with lung cancer based on the Kaplan–Meier plotter (https://kmplot.com/analysis/). **H** The correlation between the selected gene expression and survival probability in lung cancer patients treated with anti-tumor immunotherapies was analyzed according to the database based on the Kaplan–Meier plotter (https://kmplot.com/analysis/). CD274: PD-L1 gene name. NSCLC: non-small cell lung cancer. *p < 0.05

To investigate the mechanism that IFNs stimulated tumor recognition by healthy PBMCs in vitro, the differential gene expression was analyzed using a RANseq technique in A549 cells treated with 20 ng/mL of IFNγ for 2 h to discover the potential CD8^+^ T cell-recognized targets. We found 22 up-regulated (fold change > 2, *p* < 0.05) and 3 down-regulated gene expressions (fold change < 2, *p* < 0.05) in the IFNγ-treated A549 compared to parental A549 cells (Table S2). The 10 up-regulated genes with fold change > 3, including *CD274* (PD-L1), *CXCL10*, *ICAM1, BATF2, IRF1, SOCS1, HAPLN3, TAP1, PSMB9* (*LMP2*), and *MAFF*, were selected for further analysis. Consequently, qPCR was used to validate the 10 genes and another gene HLA-ABC expression in A549 cells treated with 20 ng/mL of IFNα and IFNγ. The results revealed that IFNα, IFNγ, and combined both significantly increased PD-L1, CXCL10, ICAM-1, IRF1, SOCS1, HAPLN3, TAP1, PSMB9, and MAFF levels in A549 cells (Fig. 2A). The data revealed that autocrine IFNs mediated CXCL10 expression in Fig. 1A. Moreover, we found that there were increased levels of PD-L1, ICAM-1, IRF1, and TAP1 in the RT-treated A549 cells compared to untreated A549 (Fig. 2B). The RT (10 Gy and 20 Gy)-treated A549 medium was collected and added to parental A549 cells for 2 h incubation. The 5 genes, PD-L1, CXCL10, ICAM-1, IRF1, and TAP1, were then analyzed using qPCR, which indicated that 10 Gy-treated A549 medium (10 Gy-m) significantly induced the gene expression in A549 cells (Fig. 2C). However, 20 Gy-m caused less increase in PD-L1, CXCL10, and ICAM-1 (Fig. 2C) that may be less IFNγ expression in the 20 Gy-treated A549 (Fig. 1A). In addition to confirm the observation, flow cytometry was used to validate the protein levels of PD-L1 and ICAM-1 in IFNs- and RT-treated A549 cells. We demonstrated that IFNα, IFNγ, and combined both significantly increased PD-L1 and ICAM-1 protein levels in A549 cells (Fig. 2D). Meanwhile, RT also increased PD-L1 and ICAM-1 protein levels in A549 cells in a dose-dependent manner (Fig. 2E). We further demonstrated that PD-L1 and ICAM-1 were positively correlated with each other for both mRNA and protein levels in patients with lung cancer based on the database in cBioPortal (https://www.cbioportal.org/) (Fig. 2F). To clarify the clinical significance of the selected genes, the Kaplan–Meier plotter (https://kmplot.com/analysis/) was used to analyze the correlation between the mRNA expression and survival probability in patients with lung cancer based on GEO, EGA, and TCGA databases. We found that *CD274* (PD-L1) levels by different probes presented inconsistent results, whereas *CD274* by probe 223834_at associates with a poor survival rate (*p* < 0.05) but probe 227458_at with a better survival rate (*p* < 0.05) in patients with lung cancer (Fig. 2G). In addition, *ICAM1* levels by probe 202637_s_at and 202638_s_at were associated with a better survival rate (*p* < 0.05) but probe 215485_s_at with a poor survival rate (*p* < 0.05) (Fig. 2G). For other genes, CXCL10 by probe 204533_at was correlated with a poor survival rate (*p* < 0.05) and *IRF* detected by probe 238725_at was correlated with a better survival probability in patients with lung cancer (*p* < 0.05) (Fig. 2G). To further clarify the correlation between genes and survival rate in lung cancer patients treated with immunotherapies, the Kaplan–Meier plotter (https://kmplot.com/analysis/) was used. We found that high levels of the 5 genes, PD-L1, ICAM-1, CXCL10, IRF1, and TAP1, were correlated with better survival probabilities in lung cancer patients treated with immunotherapies (*N* = 21, *p* = 0.17 for PD-L1, *p* = 0.28 for ICAM-1, *p* = 0.052 for CXCL10, *p* = 0.023 for IRF1, and *p* = 0.22 for TAP1, Fig. 2H). When selected by all tumor types treated with immunotherapies, the 5 genes were significantly correlated with better survival probabilities (*N* = 1314, *p* < 0.05, Fig. 2H).

### IFNγ mediates PD-L1 and ICAM-1 expression through the JAK2-STAT3 signaling pathway in A549

To investigate the downstream transducers involved in IFNγ-mediated gene expression, IFNγ-downstream IFNGR1, IFNGR2, JAK1, JAK2, STAT1, and STAT3 were knocked down by short hairpin RNA (shRNA) (Fig S2A, S2C, and S2E), and qPCR was used to detect PD-L1, ICAM-1, IRF1, and TAP1 in A549 treated with 20 ng/mL of IFNγ for 2 h. We found that knockdown of IFNGR1 significantly suppressed IFNγ-mediated PD-L1, ICAM-1, IRF1, and TAP1 expression (Fig S2B). Knockdown of IFNGR2 significantly suppressed IFNγ-mediated PD-L1 and ICAM-1, but increased IRF1 and TAP1 (Fig S2B). In addition, knockdown of JAK1 reduced IFNγ-mediated ICAM-1 and IRF1; knockdown of JAK2 reduced IFNγ-mediated PD-L1 and ICAM-1 (Fig S2D). Knockdown of STAT3 significantly reduced IFNγ-mediated PD-L1 and ICAM-1, but knockdown of STAT1 increased PD-L1 and ICAM-1 (Fig S2F). The results are summarized in Fig S2G, indicating that IFNγ induced PD-L1 and ICAM-1 through the IFNGR1/2-JAK2-STAT3 axis.

### Knockdown of PD-L1 increases activation of CD8^+^ T cells to suppress tumors

**Fig. 3:**
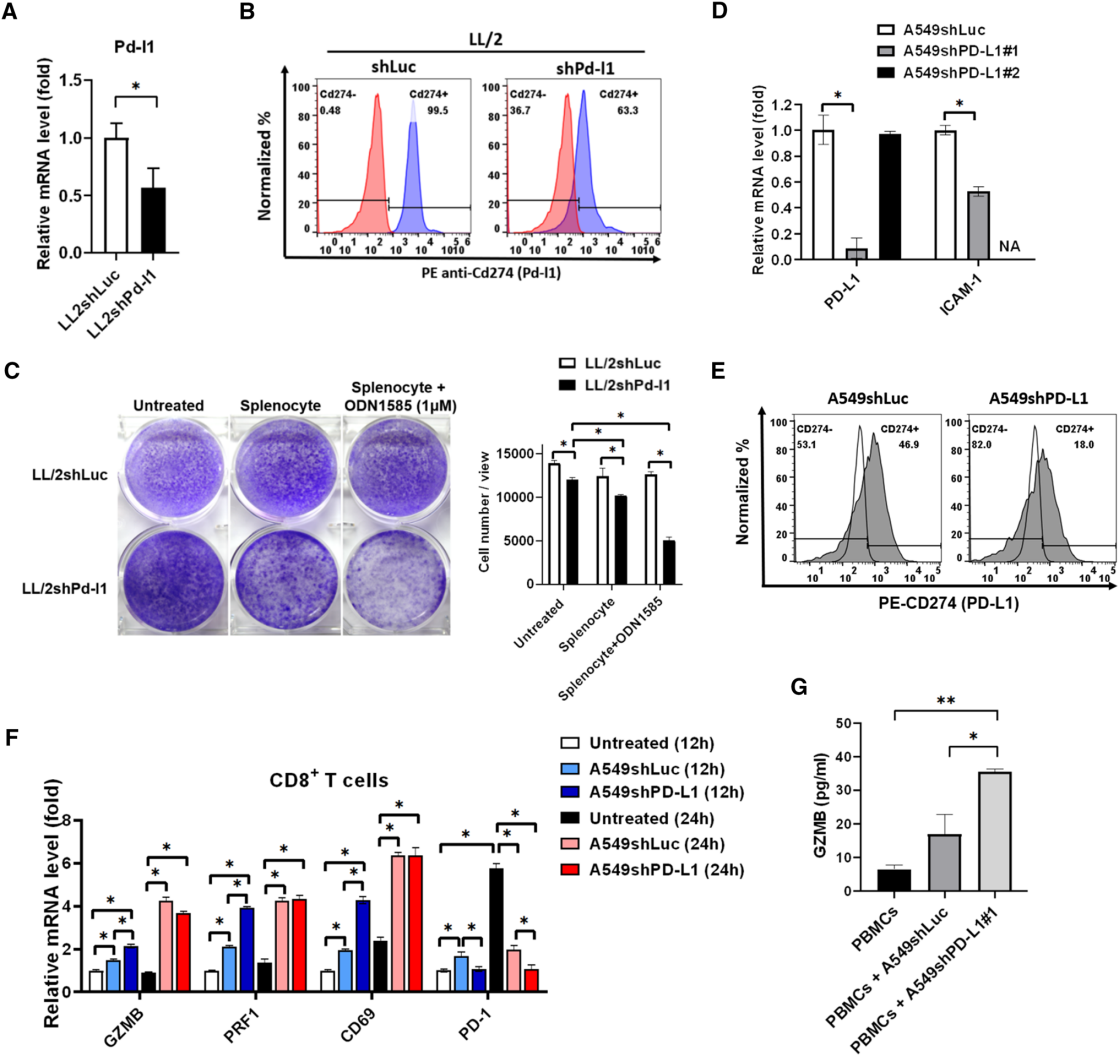
Knockdown of PD-L1 increases immunological anti-tumor efficacy and CD8^+^ T cell activation. **A** Pd-l1 was knocked down using shRNA, which was consequently validated using qPCR and (**B**) flow cytometry in LL/2 cells. **C** Colony formation was measured for assessing the splenocytes-mediated anti-tumor activity in LL/2shPd-l1 treated with or without tenfold of splenocytes and 1 μM of ODN1585 for 24 h and re-seeded and cultured for another 7-days compared to LL/2shLuc. **D** PD-L1 was knocked down in A549 cells, which was validated using qPCR and (**E**) flow cytometry. **F** Cytotoxic markers granzyme B (GZMB), perforin (PRF1), activation markers CD69, and exhaustion marker PD-1 in the isolated CD8^+^. T cells were detected using qPCR after A548shLuc and A549shPD-L1 individually treated with healthy PBMCs for 12 h and 24 h. **G** The supernatants were collected and analyzed for granzyme B expression using its specific ELISA kit after A549shLuc and A549shPD-L1 were individually treated with PBMCs for 24 h. NA: not analyzed. *p < 0.05, ** p < 0.01, ***p < 0.001

Pd-l1 was further knocked down in mouse LL/2 cells, which was validated using qPCR (Fig. 3A) and flow cytometry (Fig. 3B). We found that knockdown of Pd-l1 significantly diminished splenocytes-mediated anti-LL/2 colony formation (Fig. 3C), whereas ODN1585, a class A oligodeoxynucleotides with unmethylated CpG dinucleotides binding to Toll-like receptor 9 in plasmacytoid dendritic cells (pDCs) [35], stimulated splenocytes-mediated anti-LL/2 activity (Fig. 3C). To validate the effect of PD-L1-knockdown tumors on CD8^+^ T cell activation, PD-L1 was further knocked down in A549 cells and validated using qPCR (Fig. 3D) and flow cytometry (Fig. 3E), which were incubated with healthy PBMCs for 12 h and 24 h, and CD8^+^ T cells were isolated consequently for detection of activation and exhaustion gene expression using qPCR (Fig. 3F). We noticed that knockdown of PD-L1 with slightly decreased ICAM-1 expression in A549 cells (Fig. 3D) stimulated CD8^+^ T cells to increase cytotoxic markers GZMB and PRF1 and activation marker CD69 after 12 h incubation (Fig. 3F). Particularly, knockdown of PD-L1 decreased PD-1 expression in CD8^+^ T cells (Fig. 3F). In addition to validate the observation, ELISA was used to detect granzyme B in the co-cultured supernatant. It validated that knockdown of PD-L1 in A549 (A549shPD-L1#1) stimulated healthy PBMCs to secrete higher granzyme B compared to A549shLuc (Fig. 3G).

### Knockdown of ICAM-1 reduces PBMCs-mediated anti-A549 colony formation and CD8^+^ T cell activation

**Fig. 4:**
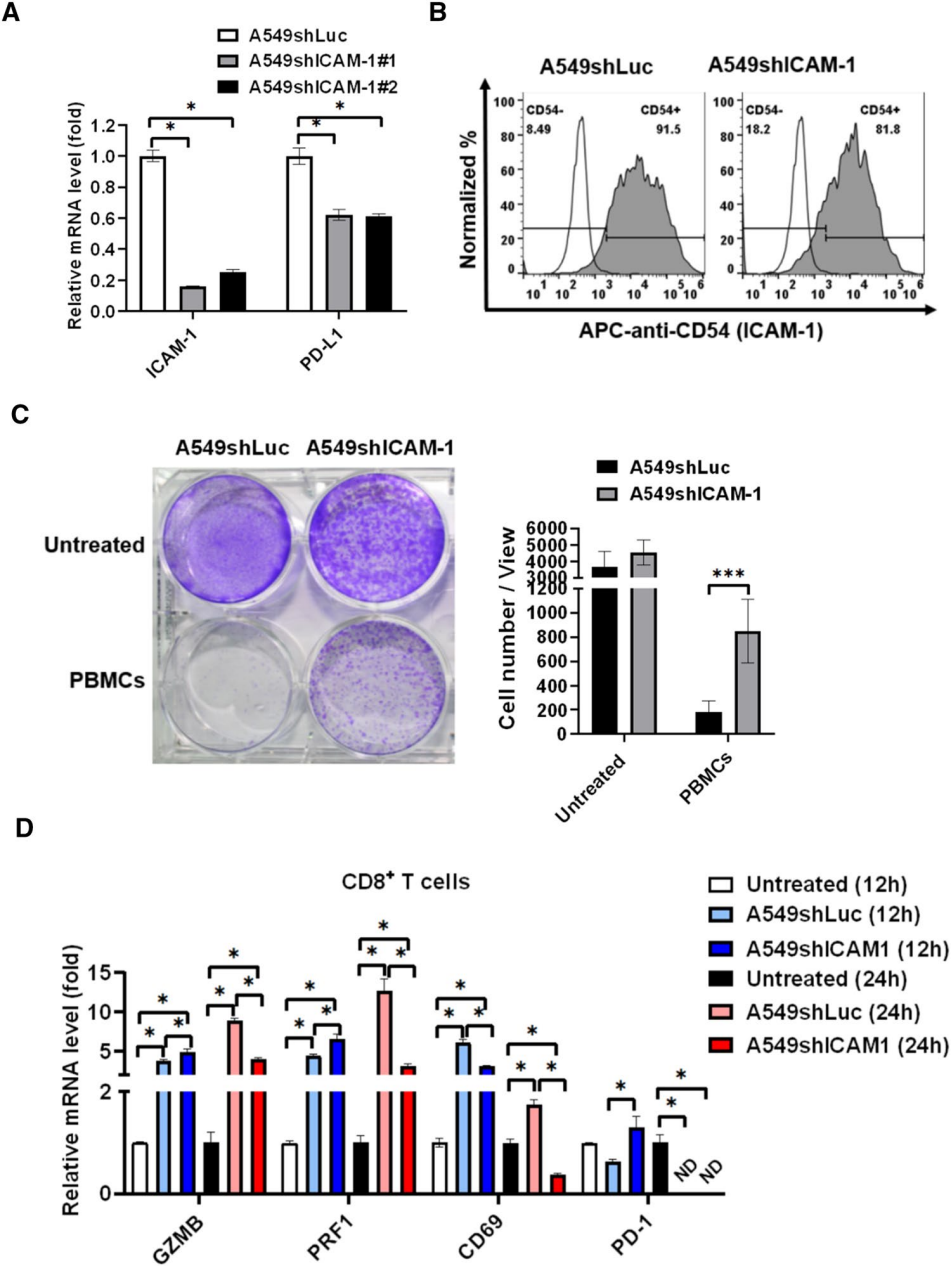
Knockdown of ICAM-1 reduces immunological anti-tumor efficacy and CD8^+^ T cell activation. **A** ICAM-1 was knocked down in A549 cells, which was validated using qPCR (**B**) and flow cytometry. Meanwhile, PD-L1 expression was also investigated in A549shICAM-1 compared to A549shLuc. **C** Colony formation was measured in A549shICAM-1 treated with healthy PBMCs for 24 h and consequently re-seeded and cultured for 7 days compared to A549shLuc. **D** Activation marker CD69, cytotoxic markers granzyme B (GZMB), perforin (PRF1), and exhaustion marker PD-1 in isolated CD8^+^. T cells were measured using qPCR after A549shLuc and A549shICAM-1 individually treated with healthy PBMCs for 12 h and 24 h. ND, non-detection. *p < 0.05, *** p < 0.001

ICAM-1 is an adhesion molecule binding to LFA-1 (CD11/CD18) for T lymphocyte transmigration [36]. To investigate the role of ICAM-1 in regulating CD8^+^ T cells, ICAM-1 was knocked down in A549 cells that were further incubated with healthy PBMCs for functional and gene expression assays. Knockdown of ICAM-1 was validated using qPCR (Fig. 4A) and flow cytometry (Fig. 4B). Meanwhile, we noticed that knockdown of ICAM-1 resulted in the down-regulation of PD-L1 in A549 cells (Fig. 4A). Nevertheless, we demonstrated that knockdown of ICAM-1 significantly reduced PBMCs-mediated anti-A549 colony formation (Fig. 4C). CD8^+^ T cells were consequently isolated and analyzed for activation, cytotoxic, and exhaustion gene expression. We found that knockdown of ICAM-1 significantly decreased A549-stimulated cytotoxic markers GZMB and PRF1 and activation marker CD69, in CD8^+^ T cells after 24 h incubation (Fig. 4D).

### CXCR3 and its binding ligand CXCL10 decreased in patients with late-stage lung cancer, contributing to the reduction in tumor-mediated immune activation

**Fig. 5:**
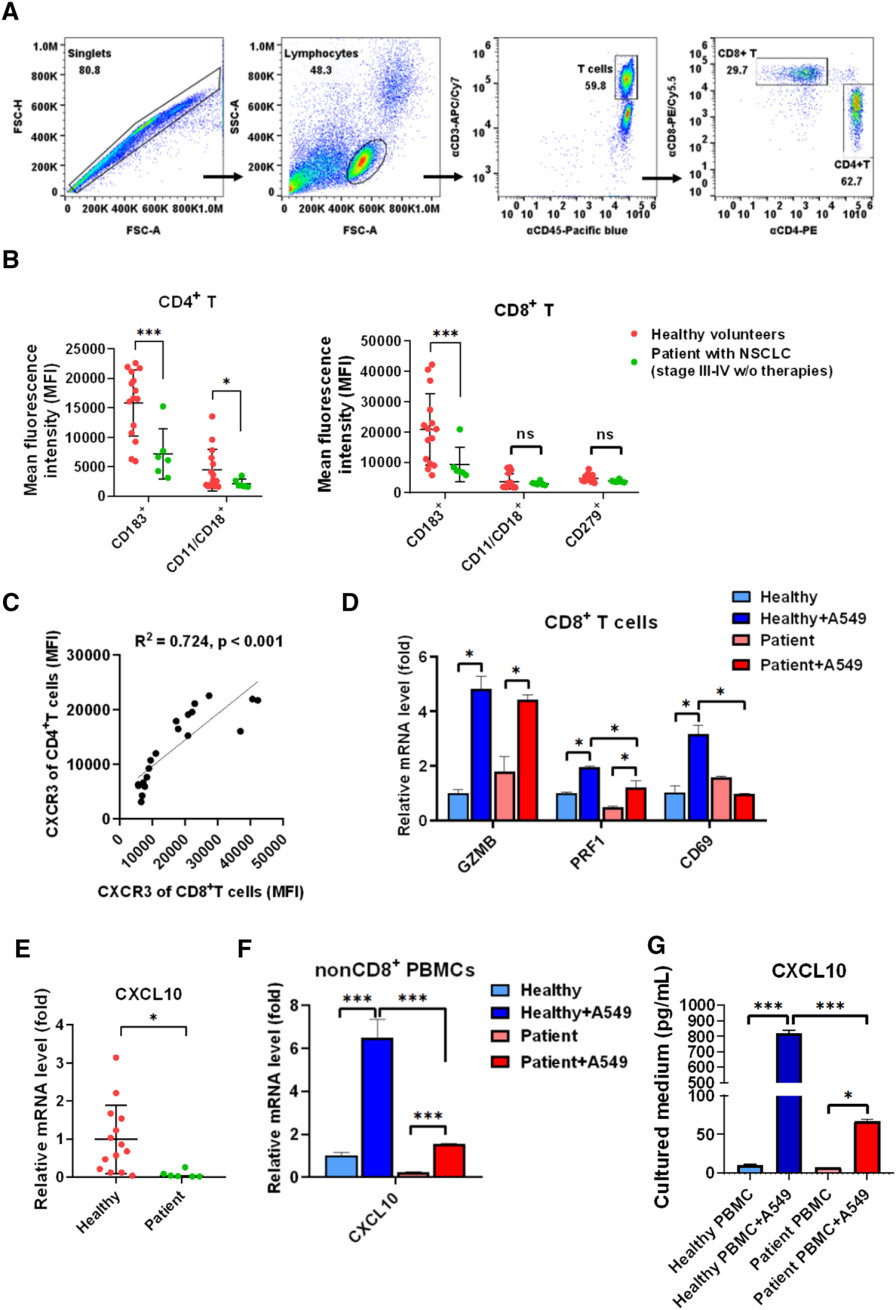
Down-regulation of CXCR3 and CXCL10 in the PBMCs of the patients with NSCLC. **A** CD4^+^ T and CD8^+^ T were distinguished by the selection of anti-CD3-APC/anti-CD45-pacific blue with consequent anti-CD4-PE and anti-CD8-PE-cy5.5 staining*.* **B** CXCR3 (CD183), LFA1 (CD11 and CD18), and PD-1 (CD279) were measured using flow cytometry in CD4^+^ T and CD8^+^ T of the enrolled healthy volunteers (*N* = 14) and patients with lung cancer (*N* = 6) (Table 1). **C** The correlation between CXCR3 expression in CD4^+^ T and CD8^+^ was analyzed using Pearson’s correlation. **D** The activation markers CD69 and cytotoxic markers granzyme B (GZMB) and perforin (PRF1) were measured using qPCR in the isolated CD8^+^ T cells after A549 was treated with PBMCs from a healthy volunteer or a patient with late-stage lung cancer. **E** CXCL10 was measured using qPCR in PBMCs from healthy volunteers (*N *= 14) and patients with lung cancer (*N* = 6), and (**F**) in nonCD8^+^. PBMCs after A549 treated with PBMCs from a healthy volunteer or a patient with late-stage of lung cancer. **G** Meanwhile, CXCL10 levels were also measured using an ELISA technique in the cultured medium after A549 was treated with PBMCs from a healthy volunteer or a patient with late-stage of lung cancer. *p < 0.05, ***p < 0.001

In an LL/2-induced tumor xenograft mouse model, we observed that RT-pretreated LL/2 mixed with parental LL/2 enhanced CD8^+^ T cell recruitment in tumor tissues detected using ^111^In-labeled αCD8 antibody in a SPECT/CT nuclear imaging platform (Fig S3). It suggests that RT enhances CD8^+^ T cell recognition of tumors that may be due to CXCL9/10-mediated recruitment of T lymphocytes [23, 37] since we demonstrated that RT also induced CXCL9/10 expression in tumor cells (Fig. 1A). To further investigate the hypothesis in clinical, we compared the difference between healthy volunteers and lung cancer patients for recognizing RT- or IFNγ-treated tumors, PD-1, a gene binding to PD-L1, CXCR3, a gene binding to CXCL10 for activating LFA-1, and LFA-1 levels were measured using flow cytometry. CD4^+^ T and CD8^+^ T were distinguished by the selection of anti-CD3-APC/anti-CD45-pacific blue with consequent anti-CD4-PE and anti-CD8-PE/Cy5.5 staining (Fig. 5A). It indicated that CD8^+^ T levels were decreased in the patients with lung cancer compared to the healthy volunteers (*p* = 0.04, Table 1). Meanwhile, we found that CXCR3 and LFA-1 decreased in CD4^+^ T cells of patients with lung cancer (N = 6) compared to healthy volunteers (N = 14) (Fig. 5B). Meanwhile, only CXCR3 decreased in CD8^+^ T cells of patients with lung cancer (Fig. 5B). CXCR3 was positively correlated between CD4^+^ T and CD8^+^ T cells (R^2^ = 0.724, *p* < 0.001, Fig. 5C). In addition, we demonstrated that CD8^+^ T cells in a patient with lung cancer exhibited lower expression of cytotoxic marker PRF1 and activation marker CD69 in co-cultured with A549 for 24 h compared to a healthy volunteer (Fig. 5D). We further demonstrated that the CXCR3 ligand CXCL10 decreased in the PBMCs of patients with lung cancer compared to healthy volunteers (Fig. 5E). Moreover, non-CD8^+^ PBMCs, containing NKs, macrophages, DCs, and CD4^+^ T cells, exhibited lower A549-stimulated CXCL10 expression in a patient with lung cancer compared to a healthy volunteer after co-cultured with A549 for 24 h (Fig. 5F). Moreover, we validated that PBMCs in a patient with lung cancer secreted less CXCL10 levels compared to healthy PBMCs in co-cultured with A549 for 24 h (Fig. 5G).

**Table 1:** Enrolled healthy volunteers and patients with lung cancer

	Healthy volunteer	Lung cancer*	*p* value
	(*N* = 14)	(*N* = 6)	
Age years, median (range)	41 ± 13	64 ± 10	
	(26–60)	(51–74)	
Gender, male (%)	8 (57.1%)	3 (50.0%)	
CD4^+^ T cell (%)	25.54 ± 11.25	34.87 ± 19.73	0.32
CD8^+^ T cells (%)	19.74 ± 8.70	11.51 ± 6.87	0.04

*Patients with late-stage lung cancer

**Fig. 6:**
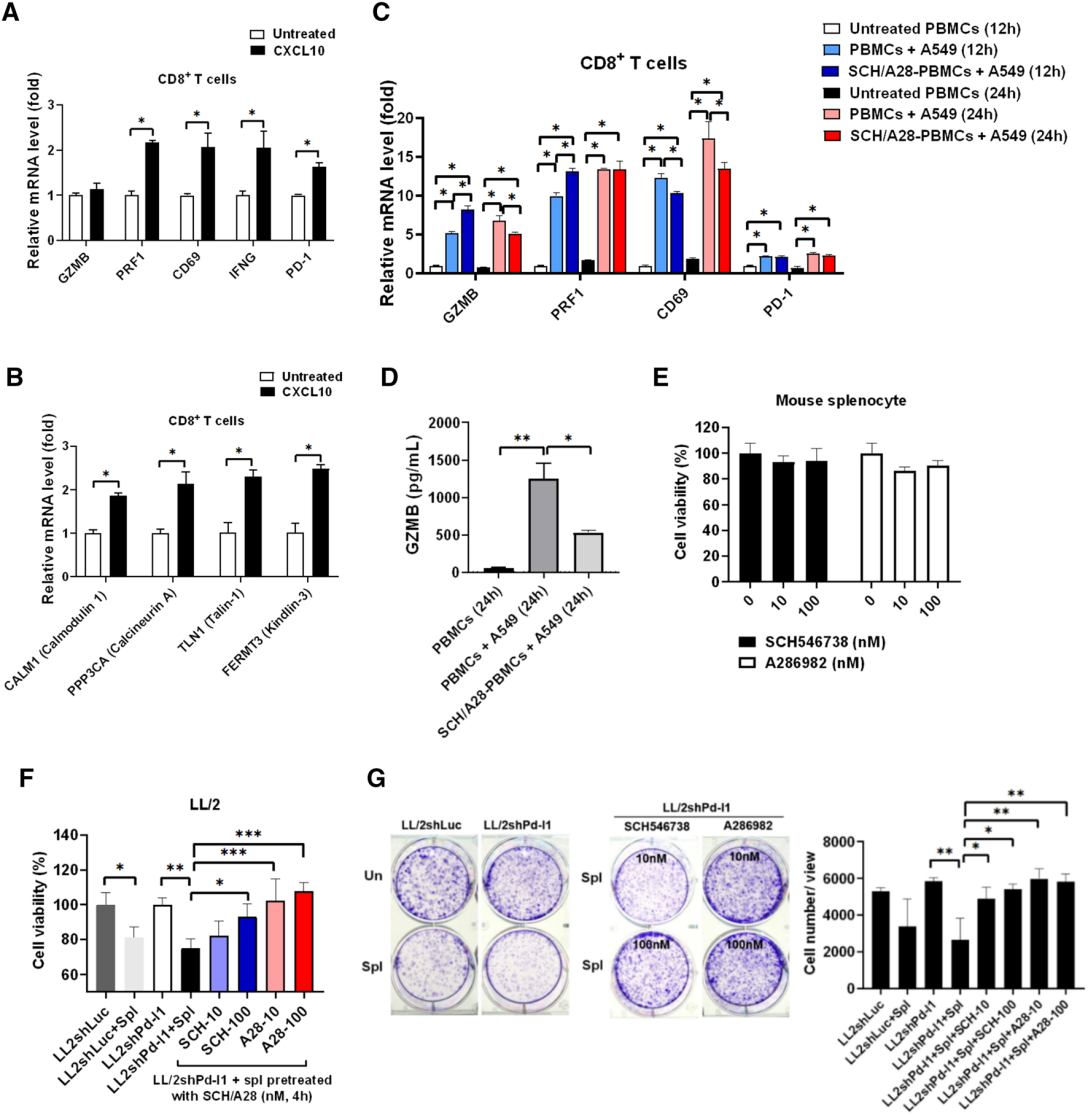
Suppression of CXCR3 and LFA-1 by SCH546738 and A286982, respectively, reduces splenocytes-mediated anti-LL/2Pd-l1 efficacy. **A** Cytotoxic marker granzyme B (GZMB), perforin (PRF1), activation marker CD69, IFNG, exhaustion marker PD-1, and (**B**) the genes involved in LFA-1 conformational activation, including Ca^2+^-binding CALM1, PPP3CA, and cytoskeletal TLN1 and FERMT3 were detected using qPCR in the isolated CD8^+^ T cells treated with 20 ng/mL of CXCL10 for 2 h. **C** In addition, GZMB, PRF1, CD69, and PD-1 in the isolated CD8^+^ T cells were detected using qPCR after healthy PBMCs and SCH546738 (SCH, 100 nM)/A286982 (A28, 100 nM)-pretreated PBMCs co-cultured with A549 for 12 h and 24 h. **D** GZMB levels in the cultured medium were measured using an ELISA kit. **E** Splenocytes, collected from a C57BL/6, which is syngeneic to LL/2 cell line, were treated with SCH546738, an inhibitor of CXCR3, and A286982, an inhibitor of ICAM-1-LFA1 interaction for 48 h. Cell viability was used to test the toxic effect of the selected compounds. **F** Cell viability and (**G**) colony formation were measured in LL/2shLuc and LL/2shPd-l1 treated with tenfold of Splenocytes (Spl), whereas splenocytes were pre-treated with or without SCH546738 (SCH-10 nM, SCH-100 nM) and A286982 (A28-10 nM and A28-100 nM) for 4 h. *p < 0.05, ** p < 0.01, ***p < 0.001

Next, we found that CXCL10 significantly increased activation markers CD69 and IFNγ (Fig. 6A) and the genes involved in LFA-1 conformational activation (Fig. 6B), including Ca^2+^-binding calmodulin 1 (CALM1), calcineurin A (PPP3CA), and cytoskeletal talin-1 (TLN1) and kindlin-3 (FERMT3) [36, 38]. The results suggested that CXCL10 triggered LFA-1 conformational activation in CD8^+^ T cells for further binding with ICAM-1 [39]. In addition, we found that SCH546738, an inhibitor of CXCR3, and A286982, an inhibitor of LFA-1, significantly reduced cytotoxic marker GZMB and activation marker CD69 expression in CD8^+^ T cells isolated from PBMCs co-cultured with A549 (Fig. 6C). ELISA results validated that PBMCs pretreated with SCH546738 and A286982 significantly reduced GZMB secretion (Fig. 6D). Further to validate CXCR3 and LFA-1 involved in CD8^+^ T cell activation, their specific inhibitors were used in splenocytes-mediated anti-LL/2shPd-l1 colony formation. We demonstrated that SCH546738, an inhibitor of CXCR3, and A286982, an inhibitor of LFA-1, did not affect the cell viability of mouse splenocytes under 100 nM concentration (Fig. 6E) but suppressed splenocytes-mediated anti-LL/2 cell viability (Fig. 6F) and colony formation (Fig. 6G) after CD8^+^ T cells isolated, incubated with inhibitors for 4 h, and re-mixed with splenocytes. The results demonstrate that CXCR3 and LFA-1 in CD8^+^ T cells play significant roles in anti-tumor immunity.

## Discussion

In this study, we validated that RT suppressed lung tumor cell proliferation by measuring tumor cell viability and colony formation. Meanwhile, we validate that RT-induced type I IFNs and ISGs expression in A549 and LL/2 lung cancer cells. Particularly, we found that RT- and IFNγ-pretreated A549 significantly induced higher CD8^+^ T cell activation compared to untreated A549 cells, resulting in higher anti-tumor immunologic activity by measurement of A549 colony formation. It is an interesting finding that IFNs-treated tumors enhance CD8^+^ T cells to recognize and suppress tumors. Through RNAseq and consequent validation using qPCR and flow cytometry, we demonstrated that RT and IFNs induced not only PD-L1 but also CXCL10 and ICAM-1. Therefore, it makes sense that PD-L1 and ICAM-1 correlatedly expressed in gene-expression (Fig. 2F) and gene-immunotherapeutic efficacy (Fig. 2H) in patients with cancers. Interestingly, PD-L1 and ICAM-1 presented opposite CD8^+^ T cell-regulatory functions; PD-L1 suppressed CD8^+^ T, but ICAM-1 activated it. These findings provide evidence and a possible mechanism that RT and IFNs enhance tumor immunotherapies in certain tumor types.

PD-L1 binds to PD-1 on CD8^+^ T cells to furthermore exhaust CD8^+^ T cells for protecting autologous cells. Therefore, PD-L1 and PD-1 are the therapeutic targets in tumor immunotherapies, such as pembrolizumab and nivolumab targeting PD-1; atezolizumab targeting PD-L1 in clinical practice. Literature has observed that overexpressed PD-L1 is highly correlated with anti-PD-1 immunotherapy [29]. However, the controversy is how PD-L1, which is already abundantly expressed in tumors, continues to express and to further increase anti-PD-1 immunotherapy. Our study demonstrated another possibility that overexpression of CXCL10 and ICAM-1 in tumor cells plays a significant role to help CD8^+^ T cell adhesion and further activation by recognizing the alloantigens in A549 cells. We notice that LL/2 cells were not suppressed by the syngeneic splenocytes from a C57BL/6 mouse; therefore, the experiment investigating the role of ICAM-1 was performed in A549-to-PBMCs considerably being allogeneic rejection. Based on the observation that RT induces PD-L1, CXCL10, and ICAM-1 at the same time, RT combined with anti-PD-1 immunotherapy is suggested in clinical practice for patients with lung cancer.

CXCR3 is a surface receptor expressed in T lymphocytes responding to T cell transmigration toward CXCL9- and CXCL10-overexpressed inflammatory tissue microenvironment [36, 40]. LFA-1 activation is mediated by CXCL9/10-CXCR3 binding and consequent Kindline-3-mediated cytoskeletal change in CD8^+^ T cells [38], which further interact with ICAM-1 expressed on the surface of tumors [41]. Specifically, we found that CXCR3 decreased in the CD4^+^ T and CD8^+^ T cells in patients with lung cancer. We speculate that low expression of CXCR3 in T lymphocytes may reduce their recruitment into the tumor microenvironment [40]. Particularly, higher tumor-infiltrated CD8^+^ T cells are correlated with better immunotherapeutic efficacy [42]. Therefore, low expression of CXCR3 of T lymphocytes in a patient with lung cancer may be a target for exchanging tumors from “cold tumors” to “hot tumors” for improving clinical immunotherapeutic efficacy. Meanwhile, we notice that CXCL10 is also low expressed in the PBMCs of patients with lung cancer compared to healthy volunteers (Fig. 5E) and in a patient’s PBMCs co-incubated with A549 cells compared to a healthy volunteer (Fig. 5F and Fig. 5G). CXCL9 and CXCL10 are secreted by DCs after phagocytosis of virus or virus-infected cells, which furthermore recruit T lymphocytes. The cGAS-STING pathway mediates induction of CXCL9/10 in DCs; therefore, STING agonists are potential as therapeutic agents [43, 44] for activating DCs and recruiting T lymphocytes in tumor treatment. We demonstrated that RT also induces CXCL9 and CXCL10 expression which was considered through activation of the cGAS-STING signaling pathway by cytosolic DNA binding to cGAS [17]. This study further demonstrated that autocrine IFNs mediated CXCL10 expression. Therefore, RT is considered for the enhancement of T lymphocytes recruitment toward the tumor microenvironment, exchanging tumor characteristics from “cold tumors” to “hot tumors.”

## Conclusion

**Fig. 7:**
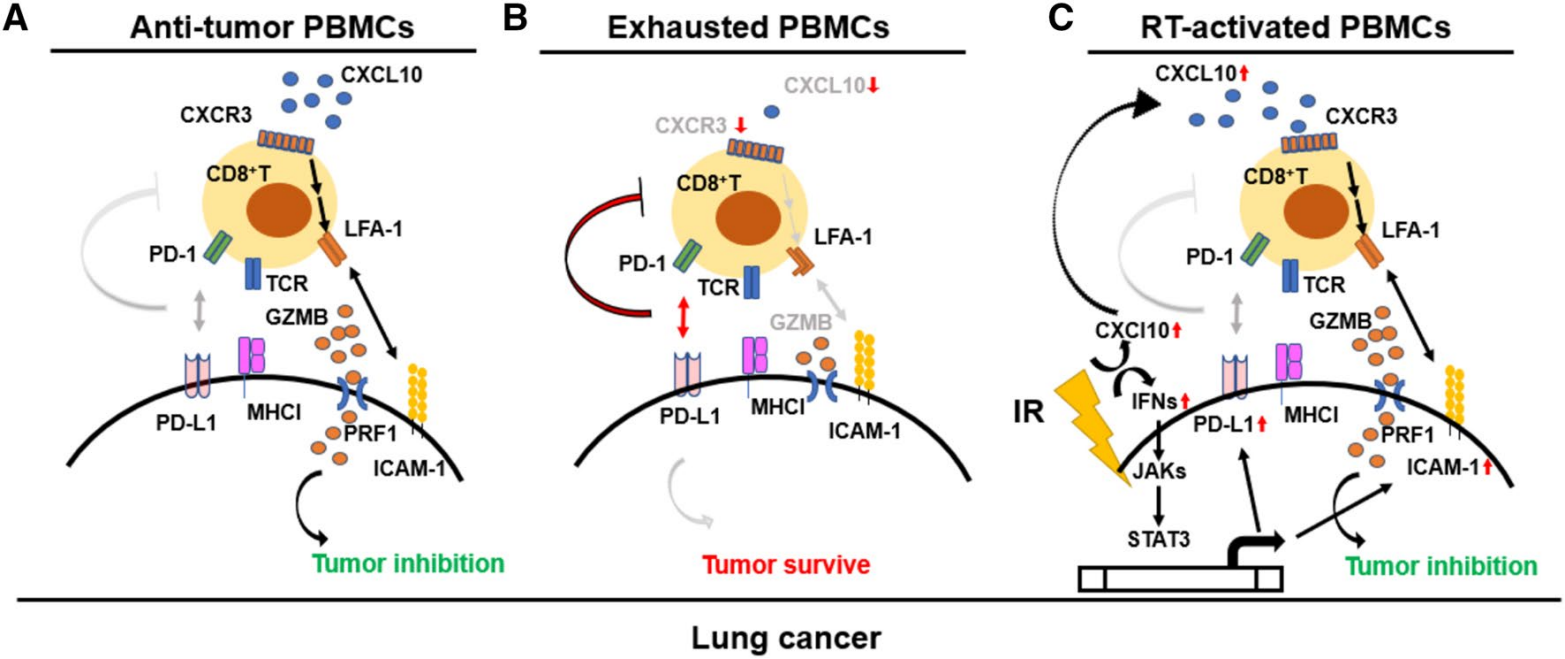
Proposed hypothesis illustrates the potential mechanism of PD-L1, CXCL10, and ICAM-1 for regulating CD8^+^ T cell activation in lung cancer. **A** CXCR3^high^CD8^+^T in healthy volunteers recognized ICAM-1 through CXCL10-activated LFA-1 to stimulate granzyme B (GZMB) and perforin (PRF1) induction in CD8^+^ T cells. High levels of CXCL10 in healthy PBMCs were considered to bind to CXCR3, resulting in activation of LFA-1 and interaction with ICAM-1. **B** Otherwise, CXCR3^low^CD8^+^T with low levels of CXCL10 in patients with lung cancer were dominantly exhausted by PD-L1, resulting in a reduction in anti-tumor immunological activity in CD8^+^ T cells. **C** irradiation (IR) induced autocrine IFNs stimulation for JAKs-STAT3 activation and consequent PD-L1 and ICAM-1 overexpression in lung cancer. Meanwhile, IR also induced CXCL10 expression in lung cancer cells to potentially activate CD8^+^ T cells through enhancing T cell adhesin with tumor cells by LFA-1-ICAM-1 interaction. RT: radiotherapy

We demonstrated that RT not only suppressed lung tumor cells in vitro but also improved PBMCs- and splenocytes-mediated anti-tumor activity. Meanwhile, IFNγ-IFNGR1/2-JAK2-STAT3 axis mediated PD-L1 and ICAM-1 expression in A549, which exhibited opposite roles for CD8^+^ T cell activation, whereas PD-L1 exhausted CD8^+^ T cells, but ICAM-1 activated it (Fig. 7). We propose that CXCR3^high^CD8^+^ T in healthy volunteers exhibited anti-tumor capacity by CXCL10/CXCR3-activated LFA-1-ICAM-1 interaction and resulted in consequent CD8^+^ T cell activation (Fig. 7A). Otherwise, CXCR3^low^CD8^+^ T in patients with lung cancer was exhausted by PD-L1 dominantly (Fig. 7B). Meanwhile, autocrine IFNs mediated CXCL10 and ICAM-1 expression in RT-treated tumors, which is potential to enhance LFA-1-ICAM-1 interaction for further activating anti-tumor CD8^+^ T cells (Fig. 7C). The study illustrates the potential mechanism of RT and IFNs in regulating CD8^+^ T cell activation in lung cancer.

### Supplementary Information

Below is the link to the electronic supplementary material.

Supplementary file1 (DOCX 1329 KB)

## Abbreviations

cGAS: Cyclic GMP-AMP synthase
DCs: Dendritic cells
DMEM: Dulbecco’s modified eagle’s medium
EGFR: Epidermal growth factor receptor
GZMB: Granzyme B
IFNα: Interferon α subtype
IFNγ: Interferon γ
IR: Irradiation
MHCI: Major histocompatibility complex class I
NSCLC: Non-small cell lung cancer
ISGs: Interferon-stimulated genes
PBMCs: Peripheral blood mononuclear cells
pDCs: Plasmacytoid dendritic cells
PD-1: Programmed cell death protein 1
PD-L1: Programmed death-ligand 1
PRF1: Perforin
qPCR: Quantitative polymerase chain reaction
RT: Radiotherapy
shRNA: Short hairpin RNA
STING: Stimulator of interferon genes
STAT1/3: Signal transducer and activator of transcription 1/3
