# Supplementary material for: Radiotherapy enhances CXCR3highCD8+ T cell activation through inducing IFNγ-mediated CXCL10 and ICAM-1 expression in lung cancer cells
Source: Cancer Immunol Immunother. 2023 Jan 23;72(6):1865–80. doi: 10.1007/s00262-023-03379-6 (PMC10198930; doi:10.1007/s00262-023-03379-6)
Supplement: Supplementary file 1 — Supplementary file1 (DOCX 1329 KB) [file 262_2023_3379_MOESM1_ESM.docx]

| **Table S1. qPCR primers** | | |
| --- | --- | --- |
| **Gene** | **Direct** | **Primer sequence** |
| *IFNA* (IFNα) | Forward | GCCATCTCTGTCCTCCATGA |
|  | Reverse | ATCTCATGATTTCTGCTCTGACAA |
| *IFNG* (IFNγ) | Forward | TCCCATGGGTTGTGTGTTTA |
|  | Reverse | AAGCACCAGGCATGAAATCT |
| *CXCL9* | Forward | TTTTCCTCTTGGGCATCATC |
|  | Reverse | TCAATTTTCTCGCAGGAAGG |
| *CXCL10* | Forward | CTGTACGCTGTACCTGCATCA |
|  | Reverse | TTCTTGATGGCCTTCGATTC |
| *ISG15* | Forward | TGTCGGTGTCAGAGCTGAAG |
|  | Reverse | GCCCTTGTTATTCCTCACCA |
| *GZMB* | Forward | ACTGCAGCTGGAGAGAAAGG |
|  | Reverse | TTCGCACTTTCGATCTTCCT |
| *PRF1* | Forward | ACTCACAGGCAGCCAACTTT |
|  | Reverse | GGGTGCCGTAGTTGGAGATA |
| *CD69* | Forward | TCTCAATGCCATCAGACAGC |
|  | Reverse | CAGTCCAACCCAGTGTTCCT |
| *PDCD1* (PD-1) | Forward | GTGTCACACAACTGCCCAAC |
|  | Reverse | CTGCCCTTCTCTCTGTCACC |
| *CD274* (PD-L1) | Forward | GTACCTTGGCTTTGCCACAT |
|  | Reverse | CCAACACCACAAGGAGGAGT |
| *ICAM1* | Forward | GGCTGGAGCTGTTTGAGAAC |
|  | Reverse | ACTGTGGGGTTCAACCTCTG |
| *BATF* | Forward | GCGAAGACCTGGAGAAACAG |
|  | Reverse | GGAGCTGACATGAGGTTGGT |
| *IRF1* | Forward | AGCTCAGCTGTGCGAGTGTA |
|  | Reverse | TAGCTGCTGTGGTCATCAGG |
| *SOCS1* | Forward | AGAGCTTCGACTGCCTCTTC |
|  | Reverse | AATCTGGAAGGGGAAGGAGC |
| *HAPLN3* | Forward | TTTGGGGAACTATCCACTGC |
|  | Reverse | TTGCCTAGGTTCTGGTCGTT |
| *TAP1* | Forward | ACGTCCACCCTGAGTGATTC |
|  | Reverse | TGGACTTTGCCAGAGATTCC |
| *PSMB9* | Forward | CATCATGGCAGTGGAGTTTG |
|  | Reverse | CAGCCAAAACAAGTGGAGGT |
| *MAFF* | Forward | TCTGTGGATCCCCTATCCAG |
|  | Reverse | CTTCTGCTTCTGCAGCTCCT |
| *HLA-ABC* | Forward | TCTACCCTGCGGAGATCACA |
|  | Reverse | TCCCATCTCAGGGTGAGGG |
| *GAPDH* | Forward | GAGTCAACGGATTTGGTCGT |
|  | Reverse | TTGATTTTGGAGGGATCTCG |
| m-*Ifna* | Forward | CAGCAGCTCAATGACCT |
|  | Reverse | GGCTGTGTTTCTTCTCTCTC |
| m-*Ifng* | Forward | ACTGGCAAAAGGATGGTGAC |
|  | Reverse | TGAGCTCATTGAATGCTTGG |
| m-*Cxcl9* | Forward | TTTTCCTCTTGGGCATCATC |
|  | Reverse | AGTCCGGATCTAGGCAGGTT |
| m-*Cxcl10* | Forward | AAGTGCTGCCGTCATTTTCT |
|  | Reverse | GTGGCAATGATCTCAACACG |
| m-*Isg15* | Forward | AAGAAGCAGATTGCCCAGAA |
|  | Reverse | TCTGCGTCAGAAAGACCTCA |
| m-*Pdl1* | Forward | CTGCCAAAGGACCAGCTTTT |
|  | Reverse | GGCTGGATCCACGGAAATTC |
| m-*Gapdh* | Forward | GGGTCCCAGCTTAGGTTCAT |
|  | Reverse | CATTCTCGGCCTTGACTGTG |
| m: mouse gene. | | |

| **Table S2. RNAseq revealed the IFNγ-mediated differential gene expression in A549 cells.** | | | | | | |
| --- | --- | --- | --- | --- | --- | --- |
| **Gene ID** | **Gene name** | **Expression** | | **Fold Change (Log2)** | **Regulation** | **P value** |
|  |  | **A549** | **A549 + IFNγ** |  |  |  |
| 29126 | *CD274** | 0.05 | 5.95 | 6.894818 | Up | 1.59E-66 |
| 3627 | *CXCL10** | 0.01 | 0.08 | 6.321928 | Up | 0.000241086 |
| 3383 | *ICAM1* | 1.06 | 42.59 | 5.328379 | Up | 0 |
| 116071 | *BATF2* | 0.78 | 30.44 | 5.28635 | Up | 3.52E-197 |
| 3659 | *IRF1* | 11.45 | 291.45 | 4.669829 | Up | 0 |
| 8651 | *SOCS1* | 0.87 | 21.4 | 4.620452 | Up | 6.94E-78 |
| 145864 | *HAPLN3* | 5.87 | 72.96 | 3.635673 | Up | 0 |
| 6890 | *TAP1* | 8.92 | 96.68 | 3.438102 | Up | 0 |
| 5698 | *PSMB9* | 2.82 | 29.55 | 3.389391 | Up | 3.43E-73 |
| 23764 | *MAFF* | 3.8 | 39.79 | 3.388335 | Up | 9.26E-217 |
| 6347 | *CCL2* | 100.35 | 791.62 | 2.979767 | Up | 0 |
| 9235 | *IL32* | 6.97 | 49.21 | 2.819721 | Up | 2.03E-90 |
| 23780 | *APOL2* | 10.58 | 73 | 2.786557 | Up | 0 |
| 6737 | *TRIM21* | 11.97 | 77.97 | 2.703496 | Up | 1.21E-287 |
| 80830 | *APOL6* | 1.59 | 10.34 | 2.701138 | Up | 3.69E-204 |
| 3437 | *IFIT3* | 2.46 | 15.6 | 2.664816 | Up | 2.27E-74 |
| 10379 | *IRF9* | 9.78 | 57.66 | 2.559664 | Up | 7.19E-175 |
| 80271 | *ITPKC* | 4.91 | 25.56 | 2.380093 | Up | 8.45E-145 |
| 83666 | *PARP9* | 2.89 | 14.84 | 2.36035 | Up | 3.59E-101 |
| 3133 | *HLA-E* | 23.73 | 116.58 | 2.296536 | Up | 0 |
| 4665 | *NAB2* | 10.56 | 44.5 | 2.075196 | Up | 3.02E-170 |
| 8542 | *APOL1* | 4.09 | 16.57 | 2.018401 | Up | 1.02E-65 |
| 1958 | *EGR1* | 73.78 | 13.9 | -2.40814 | Down | 0 |
| 10140 | *TOB1* | 64.09 | 12.48 | -2.36048 | Down | 2.22E-229 |
| 2353 | *FOS* | 58.23 | 13.37 | -2.12276 | Down | 6.42E-179 |
| *Expression of A549 + IFNγ > 10 was selected and listed in this table but the gene associated with immunotherapies was particularly selected and presented. | | | | | | |


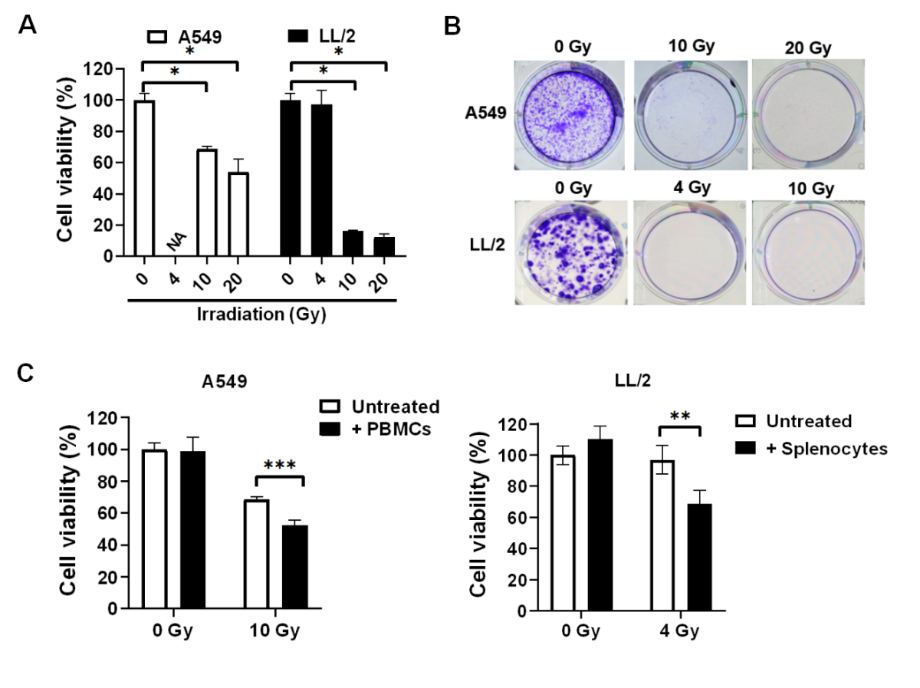


**Figure S1. Radiotherapy suppresses lung cancer cells and enhances immunological anti-tumor efficacy *in vitro.*** (A) Cell viability was measured for the human A549 and mouse LL/2 cell lines treated with irradiation and post continuous 48 h cell culture. (B) Meanwhile, colony formation was measured in A549 and LL/2 treated with irradiation and post continuous 7 days cell culture. (C) A549 and LL/2 cells treated with or without 10-fold PBMCs and splenocytes, respectively, for 24 h incubation were re-seeded and cultured for another 48 h. Cell viability was then used to assess the anti-tumor activity of PBMCs or splenocytes. **p* < 0.05, ***p* < 0.01*, ***p* < 0.001*.*


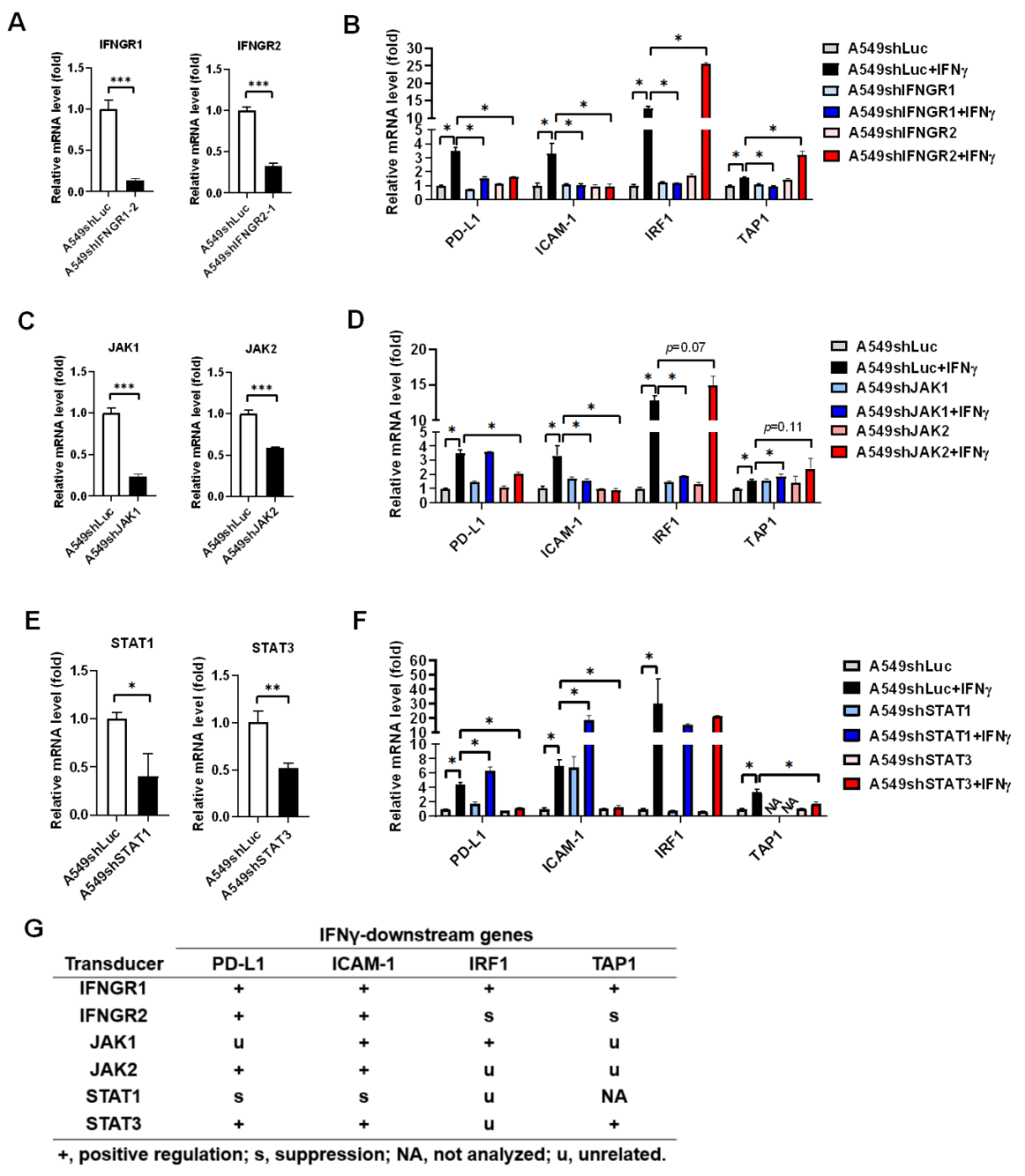


**Figure S2. IFNγ increases PD-L1 and ICAM-1 through the JAK2-STAT3 signaling pathway in A549 cells.** (A) IFNGR1 and IFNGR2 were knocked down using an shRNA technique that was validated using qPCR. (B) The expression of the four IFNγ-mediated genes (PD-L1, ICAM-1, IRF1, TAP1) was detected using qPCR in A549shIFNGR1 and A549shIFNGR2 treated with 20 ng/mL of IFNγ for 2 h compared to A549shLuc. (C) JAK1 and JAK2 were knocked down and validated using qPCR. (D) The four IFNγ-mediated genes (PD-L1, ICAM-1, IRF1, TAP1) were detected using qPCR in A549shJAK1 and A549shJAK2 compared to A549shLuc. (E) STAT1 and STAT3 were knocked down and validated using qPCR. (F) The four IFNγ-mediated genes (PD-L1, ICAM-1, IRF1, TAP1) were detected using qPCR in A549shSTAT1 and A549shSTAT3 compared to A549shLuc. (G) The results for the transducers involved in regulating IFNγ-downstream PD-L1, ICAM-1, IRF1, and TAP1 were summarized. **p* < 0.05*, **p* < 0.01, ****p* < 0.001*.*


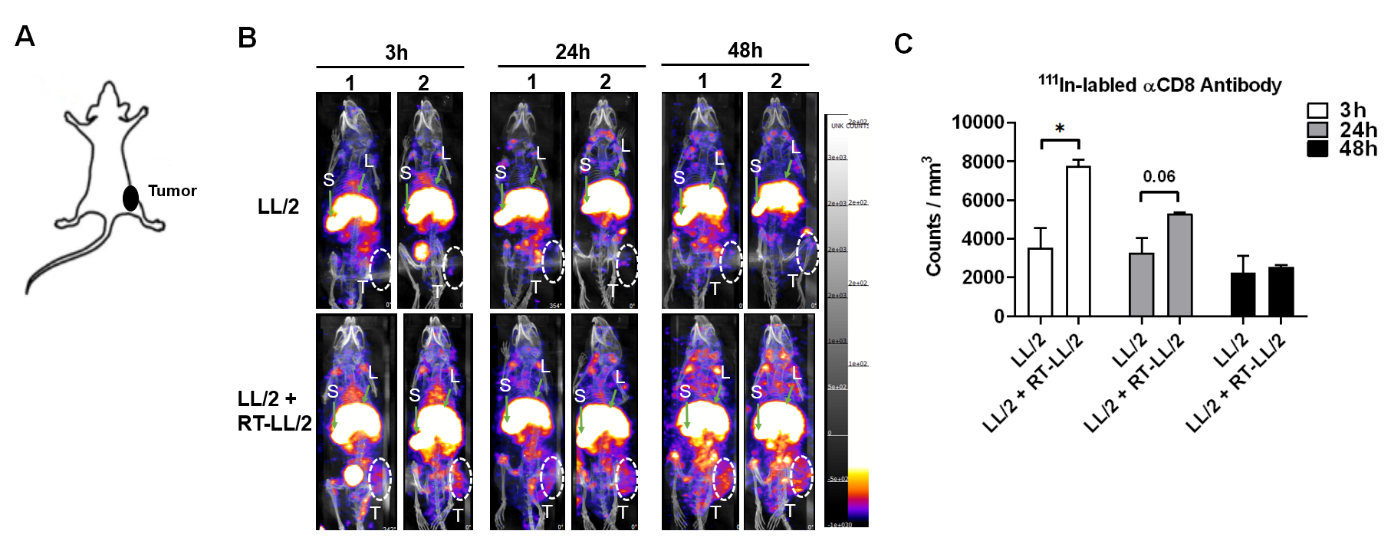


**Figure S3. Radiotherapy (RT)-treated LL/2 mixed with LL/2 enhances CD8^+^ T cell recruitment towards tumor microenvironment detected using ^111^In-labled αCD8 antibody nuclear imaging platform.** (A) Mouse LL/2 lung tumor cells were injected into the subcutaneous legs of mice of 5 weeks old C57BL/6 mice to establish the tumor xenograft model. (B) A PET/SPECT nuclear imaging platform was used to detect the injected ^111^In-labled αCD8 antibody in the LL/2 and LL/2 mixed with 10 Gy RT-LL/2 tumor xenografts. (C) Quantification of radioactive counts in tumors was investigated and compared. **p* < 0.05. T: tumor; S: spleen; L: liver.
